# Supplementary material for: Nuclear and cytoplasmic specific RNA binding proteome enrichment and its changes upon ferroptosis induction
Source: Nat Commun. 2024 Jan 29;15:852. doi: 10.1038/s41467-024-44987-9 (PMC10825125; doi:10.1038/s41467-024-44987-9)
Supplement: Supplementary file 1 — Supplementary Information [file 41467_2024_44987_MOESM1_ESM.pdf]

Supporting Information

Nuclear and Cytoplasmic Specific RNA Binding Proteome Enrichment and Its Changes upon Ferroptosis Induction

Haofan Sun [1], Bin Fu[1], Xiaohong Qian[1], Ping Xu[1] and Weijie Qin[1, 2, *]

[1] State Key Laboratory of Medical Proteomics, Beijing Proteome Research Center, National Center for Protein Sciences (Beijing), Beijing Institute of Lifeomics, Beijing 102206, China

[2] College of Chemistry and Materials Science, Hebei University, Baoding, 071002, China

* Corresponding author email: aunp_dna@126.com (W.Q.)

Supplementary Figures

a

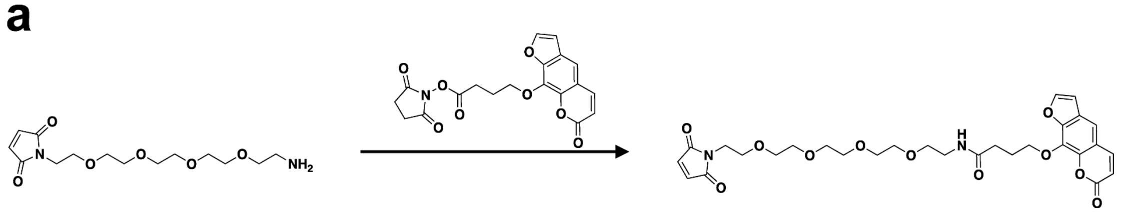

b

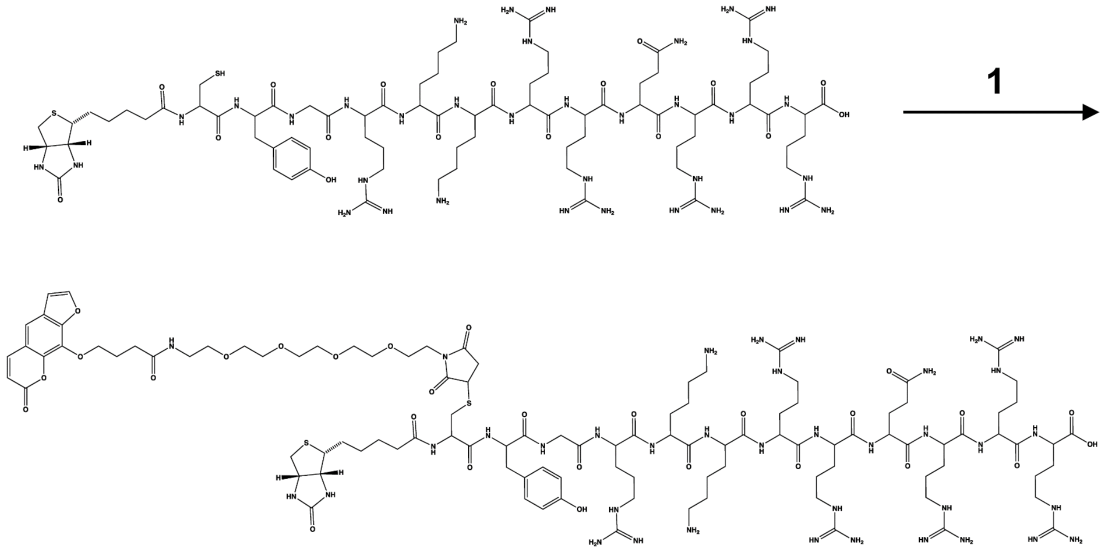

BTF

c

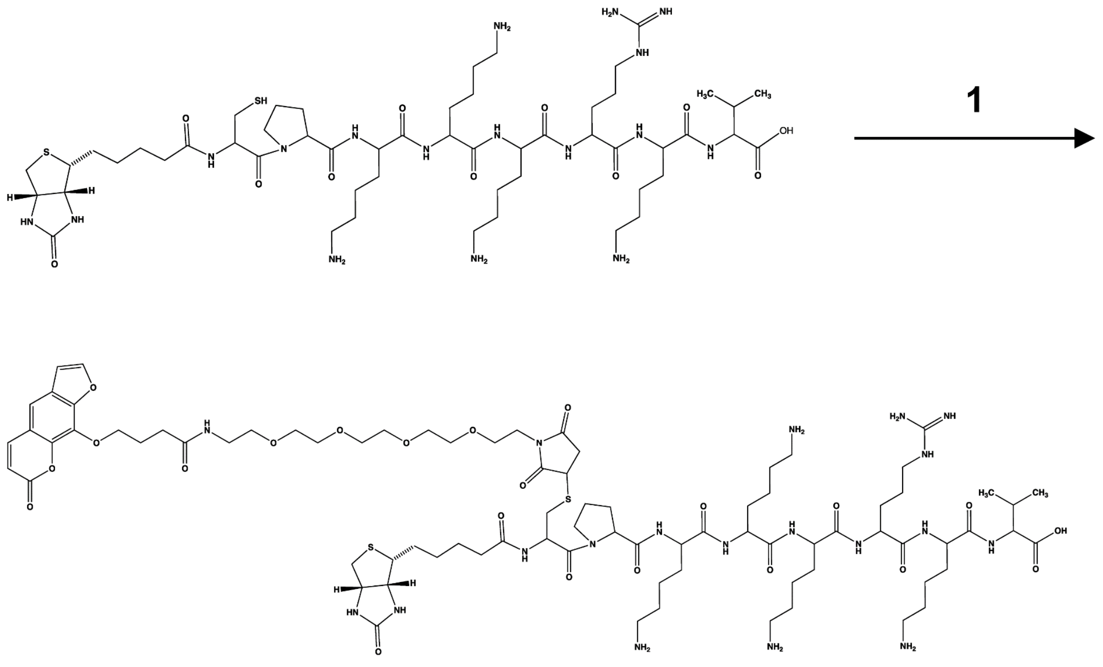

BLF

d

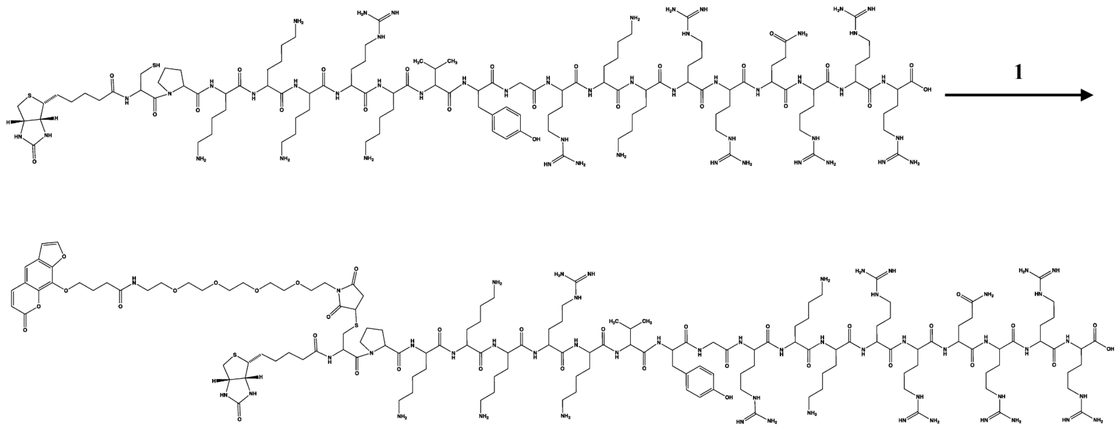

BLTF

e

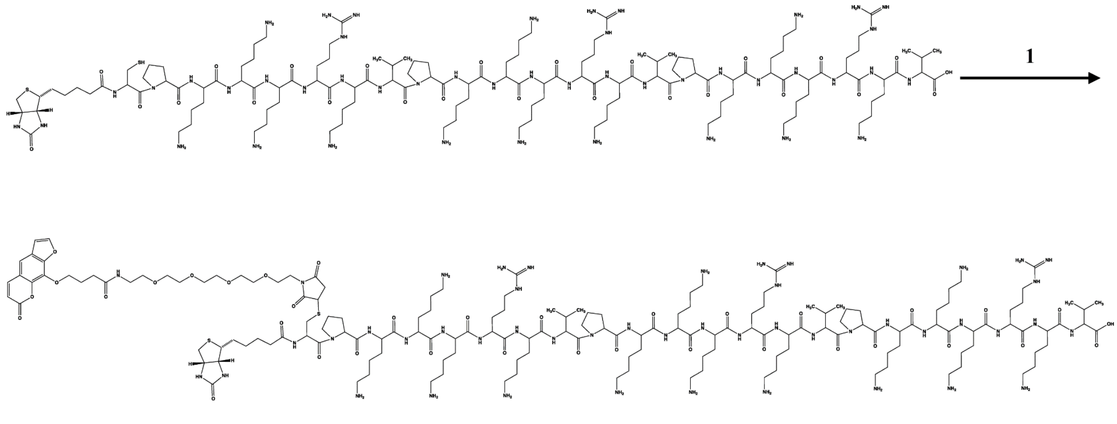

BL3F

f

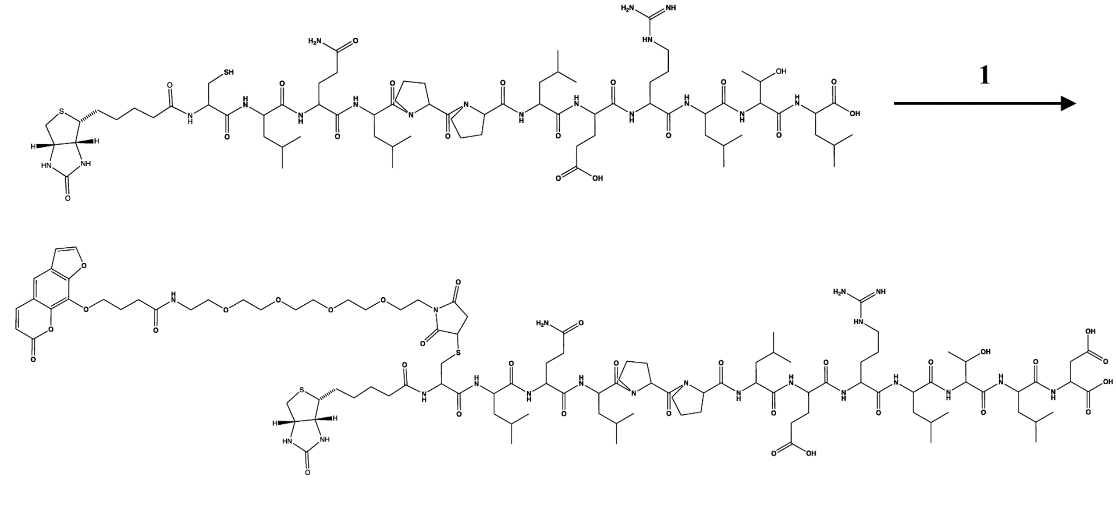

BEF

g

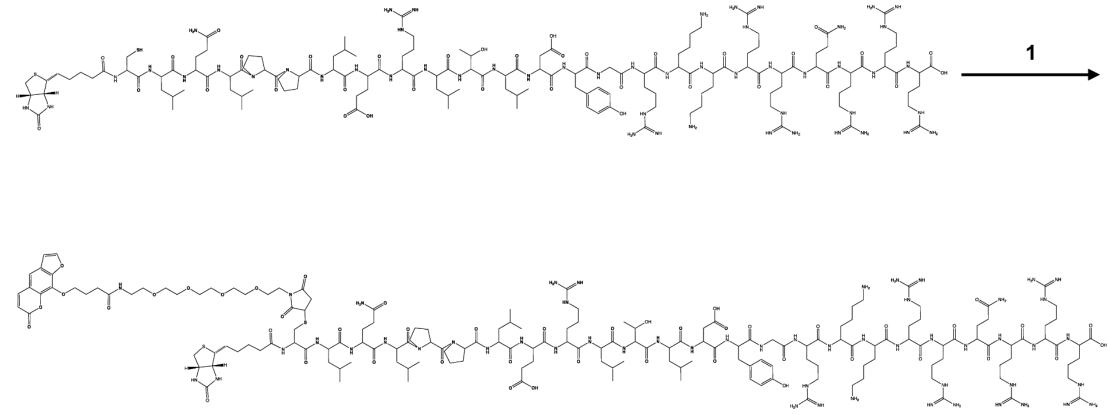

BETF

h

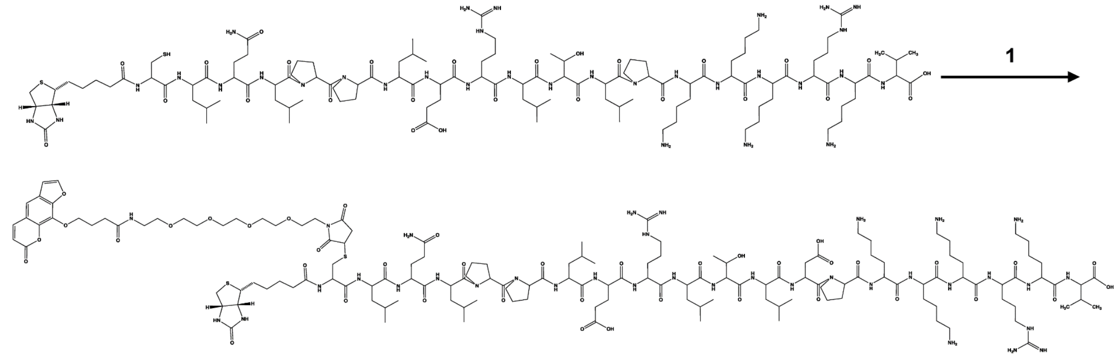

BELF

i

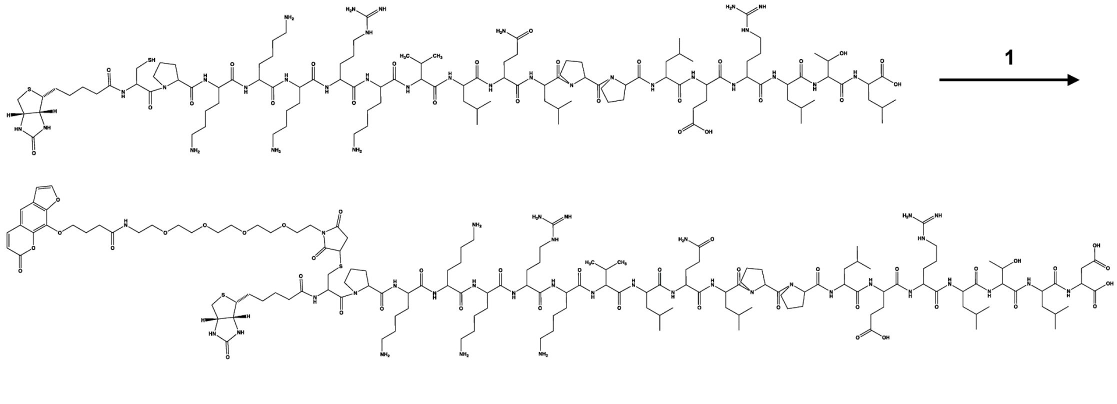

BLEF

Figure S1. Synthetic routes of the subcellular targeting probes.

a-i Synthesis of (**a**) N-(14-(2,5-dioxo-2,5-dihydro-1H-pyrrol-1-yl)-3,6,9,12-tetraoxatetradecyl)-4-((7-oxo-7H-furo[3,2-g]chromen-9-yl)oxy)butanamide (**1**), (**b**) Biotin-C(Furocoumarin)YGRKKRRQRRR (Biotin-TAT- Furocoumarin, BTF), (**c**) Synthesis of Biotin-C(Furocoumarin)PKKKRKV (Biotin-NLS- Furocoumarin, BLF),

(**d**) Biotin-C(Furocoumarin)PKKKRKVYGRKKRRQRRR (Biotin-NLS-TAT-Furocoumarin, BLTF), (**e**) Biotin-C(Furocoumarin)PKKKRKVPKKKRKVPKKKRKV (Biotin-NLS-NLS-NLS-Furocoumarin, BL3F), (**f**) Biotin-C(Furocoumarin)LQLPPLERLTLD (Biotin-NES-Furocoumarin, BEF), (**g**) Biotin-C(Furocoumarin)LQLPPLERLTLDYGRKKRRQRRR (Biotin-NES-TAT-Furocoumarin, BETF), (**h**) Biotin-C(Furocoumarin)LQLPPLERLTLDPKKKRKV (Biotin-NES-NLS- Furocoumarin, BELF) and (**i**) Biotin-C(Furocoumarin) PKKKRKVLQLPPLERLTLD (Biotin-NLS-NES- Furocoumarin, BLEF).

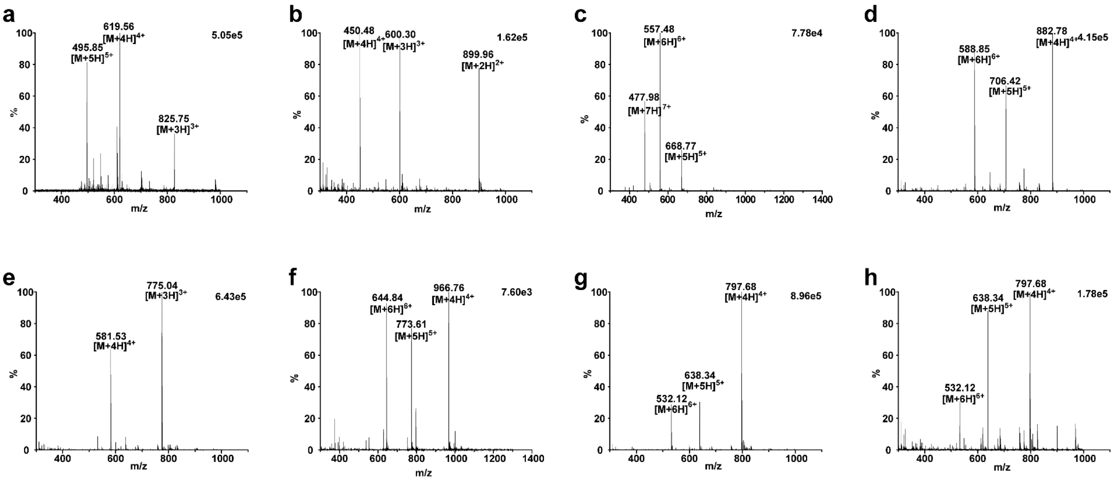

Figure S2. MS spectra of the synthesized probes.

a-h MS spectra of (**a**) BTF, (**b**) BLF, (**c**) BLTF, (**d**) BL3F, (**e**) BEF, (**f**) BETF, (**g**) BELF and (**h**) BLEF. Detailed information was listed in Table S1.

Table S1. List of theoretical and measured molecular weights of the synthesized probes.

	Probe	Theoretical molecular weight (Da)	Measured molecular weight (Da)
A	BTF	2474.25	2474.25
B	BLF	1797.91	1797.91
C	BLTF	3338.86	3338.86
D	BL3F	3527.12	3527.12
E	BEF	2322.12	2322.12
F	BETF	3863.06	3863.06
G	BELF	3186.72	3186.72
H	BLEF	3186.72	3186.72

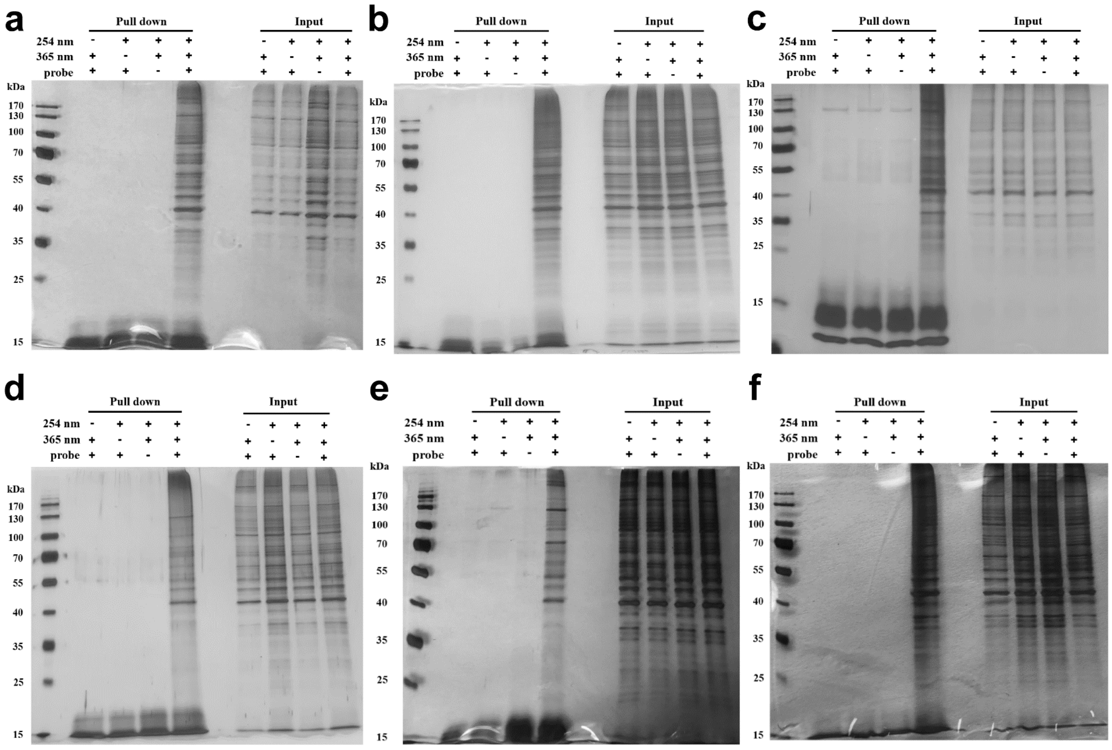

Figure S3. SDS-PAGE characterization of furocoumarin probes enriched RBPs.

a-f SDS-PAGE characterization of (**a**) BLF, (**b**) BLTF, (**c**) BL3F, (**d**) BETF, (**e**) BELF, (**f**) BLEF enriched RBPs.

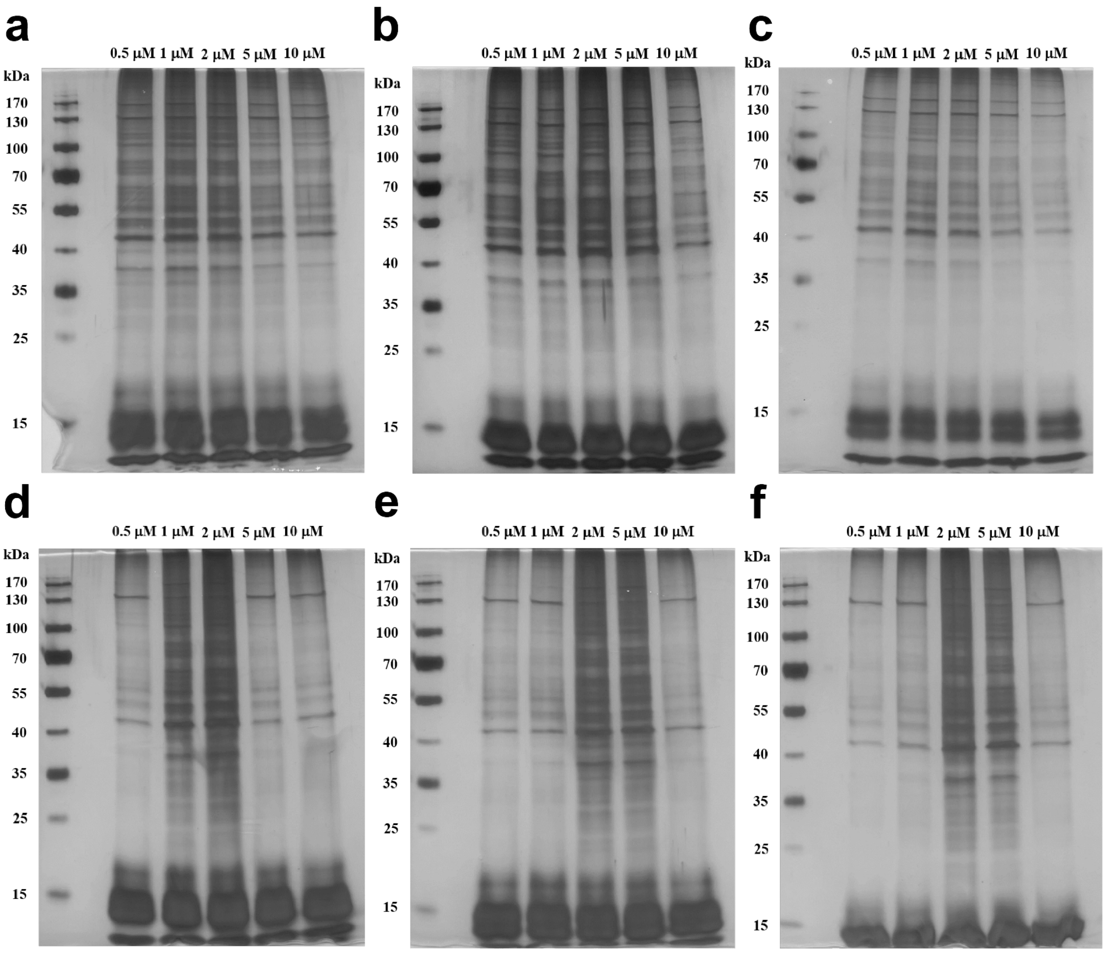

Figure S4. Optimization of the furocoumarin probes tagging concentration.

a-f Optimization of the (**a**) BLF, (**b**) BLTF, (**c**) BL3F, (**d**) BETF, (**e**) BELF, (**f**) BLEF tagging concentration.

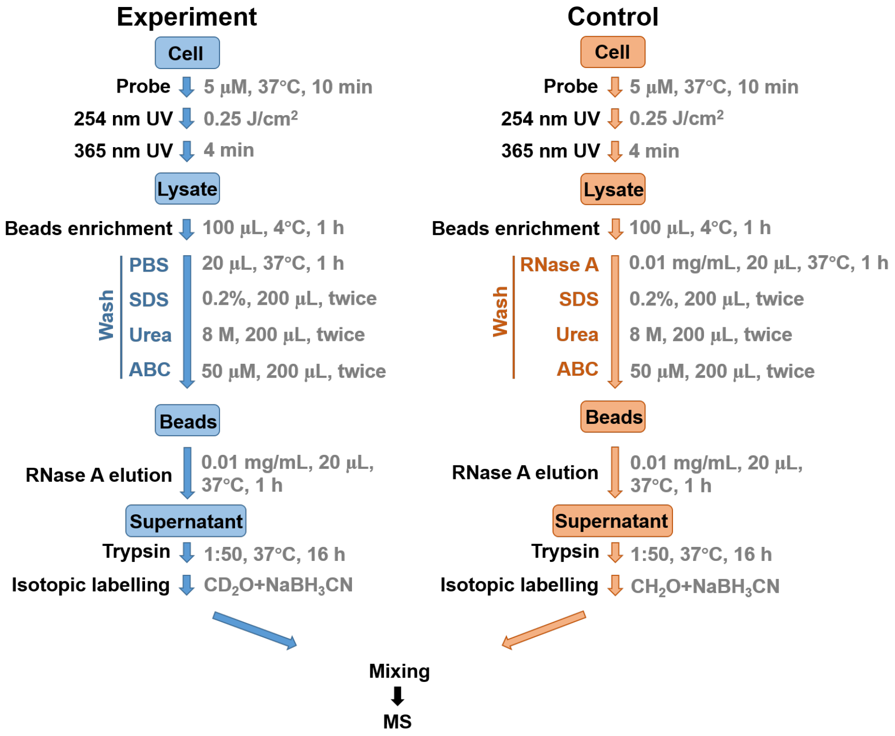

Figure S5. Detailed experimental design of the quantitative differential proteomic comparison between the experimental group and control group for RBP identification.

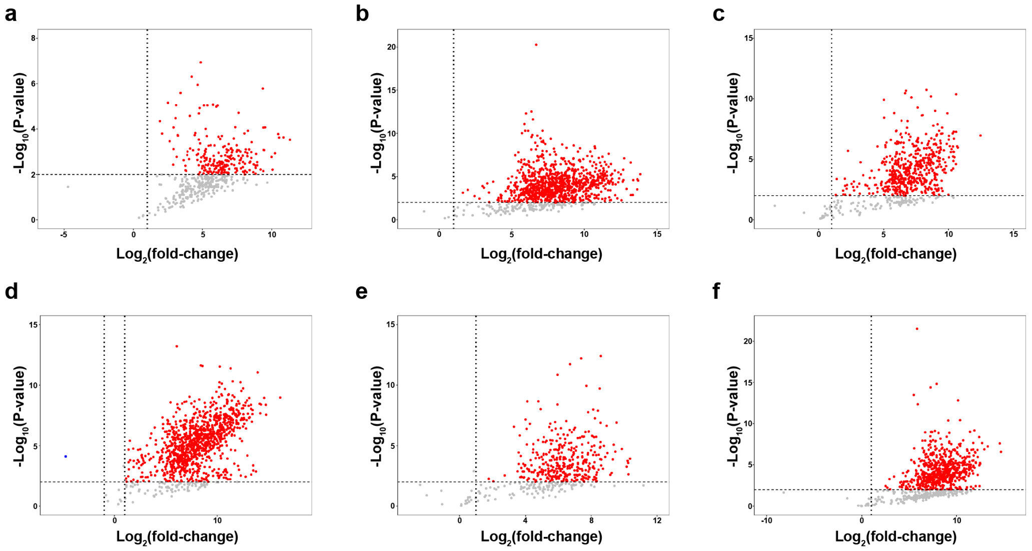

Figure S6. Scatter plot for RBPs identification by quantitative differential proteomic comparison.

a-f Scatter plot of (**a**) BLF, (**b**) BLTF, (**c**) BL3F, (**d**) BETF, (**e**) BELF, (**f**) BLEF displaying the $\log_2$ fold change (x-axis) and -log P values (y-axis) for RBPs identification by quantitative differential proteomic comparison between the experiment group and control group. Red dots represented the identified RBPs with a stringent screening cut-off. Statistical analysis was performed with two-sided Student's t-test (Benjamini&Hochberg (BH) adjusted P values) from three biological replicates.

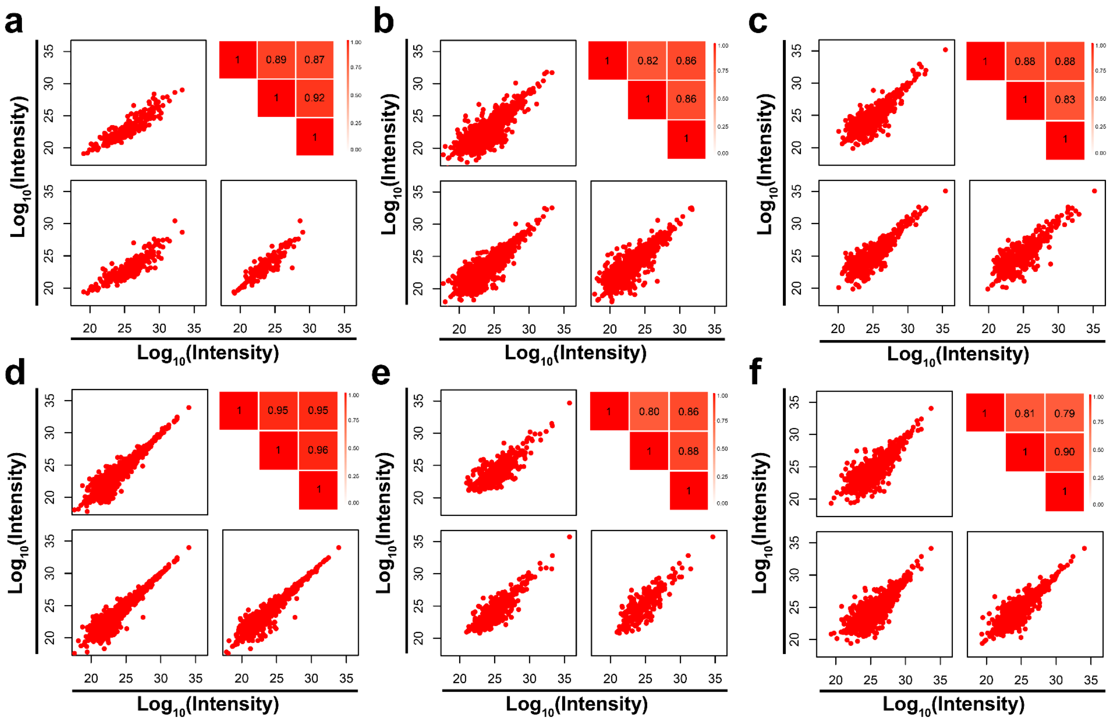

Figure S7. Reproducibility evaluation of the RBPs enriched by the furocoumarin probes. **a-f** Reproducibility evaluation of the RBPs enriched by (**a**) BLF, (**b**) BLTF, (**c**) BL3F, (**d**) BETF, (**e**) BELF, (**f**) BLEF and identification by mass spectrometry in three technical replicates.

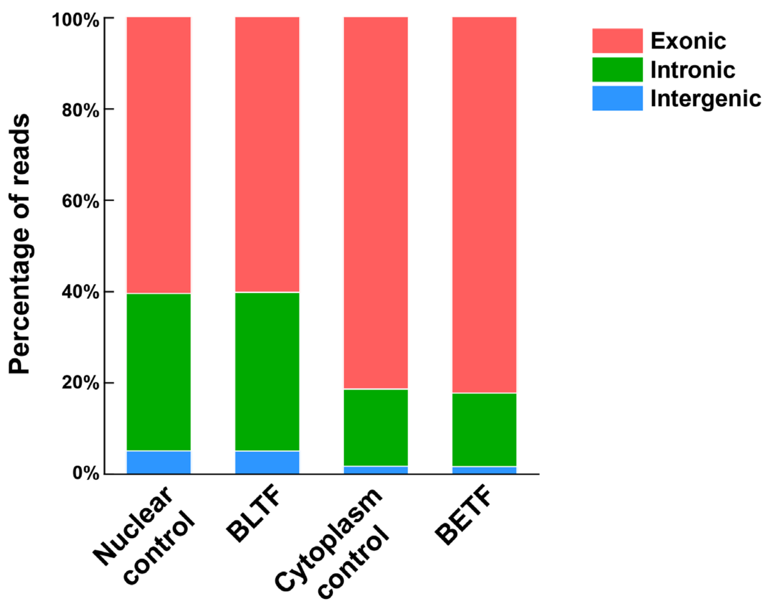

Figure S8. RNA-seq quality control metrics by reads counting.

Intronic regions are enriched in the nuclear RNA isolated by BLTF. Intergenic regions accounted for only about 5% and 1% of reads in the enrichment result of BLTF and BETF, indicating relatively low levels of DNA contamination. Three biological replicates were repeated with similar results.

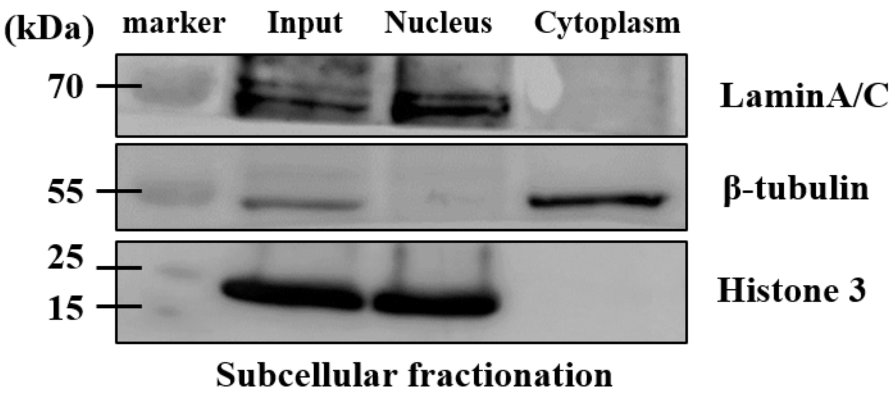

Figure S9. Validation of subcellular fractionation by western blotting.
Lamin A/C and histone 3 were used as nuclear markers and β-tubulin was used as the cytoplasmic maker.

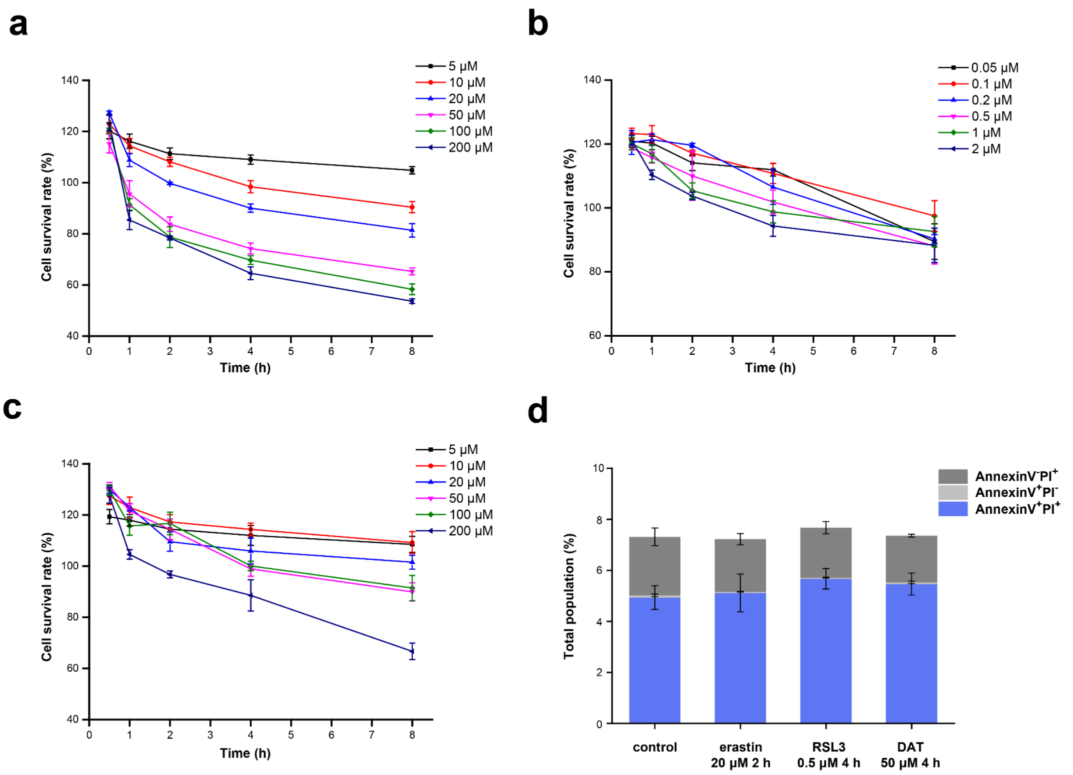

Figure S10. Biocompatibility of Hela cells treated with ferroptosis inducers. Biocompatibility of Hela cells after treating with (**a**) erastin, (**b**) RSL3 and (**c**) DAT of different concentrations and incubation times. The cell survival rate was analyzed by the CCK-8 assay.

d Ferroptotic cell death measured by flow cytometry of the cells.

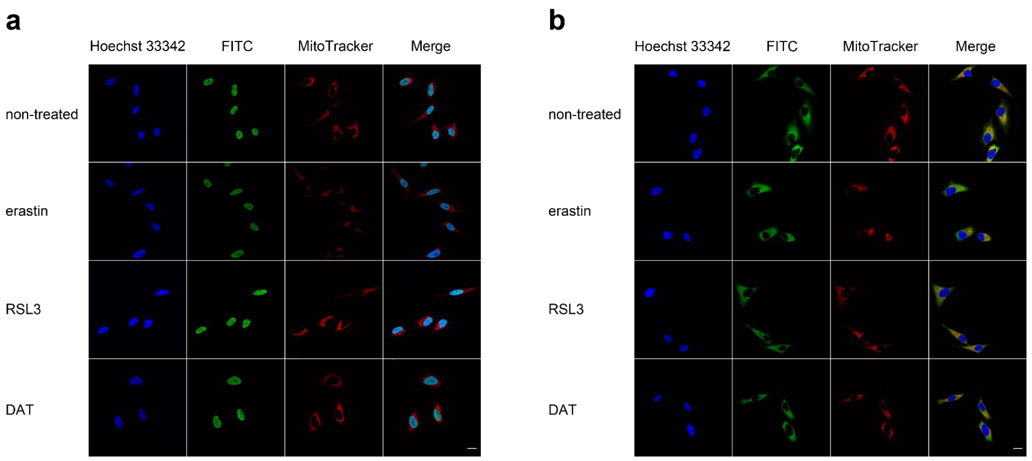

Figure S11. Confocal fluorescent imaging of probes with and without ferroptosis inducer treatment.

a, b The (**a**) BLTF and (**b**) BETF probes were stained with FITC (green) via biotin and streptavidin coupling. Hoechst 33342 (blue) was used as a nuclear marker and Mitotracker (red) was used as a mitochondria maker. Scale bars, 20 μm.

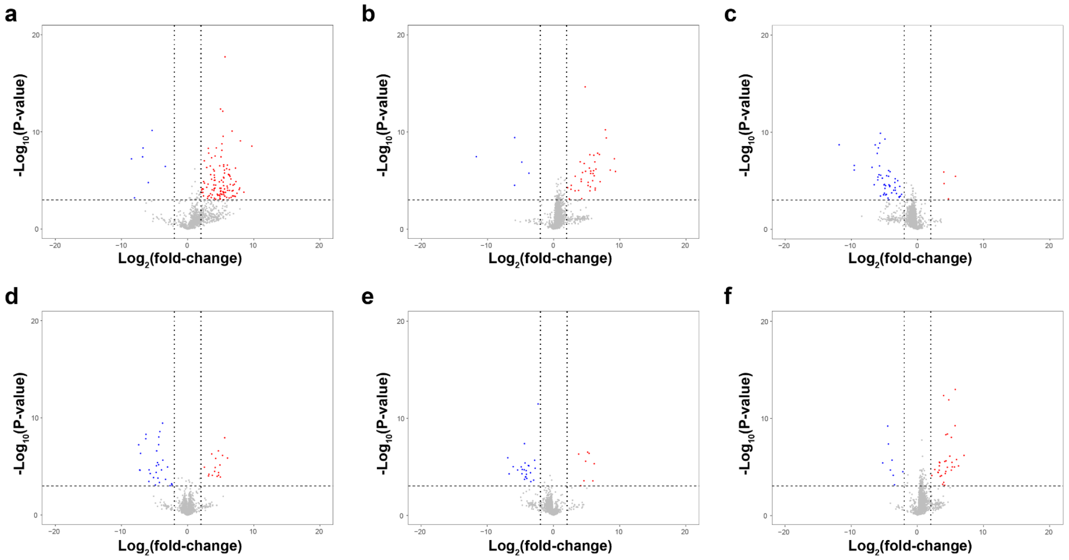

Figure S12. Quantitative analysis of nuclear/cytoplasmic proteins in cells treated with or without ferroptosis inducer.

a-c Volcano plot displaying $\log_2$ fold change (FC) (x-axis) and $-\log_{10}$ P values (y-axis) of the nuclear proteome of the (**a**) erastin-, (**b**) RSL3-, (**c**) DAT-treated and nontreated cells. Proteins with significant increase after treatment were depicted in red, and those significantly decreased after erastin treatment were depicted in blue.

d-f Volcano plot displaying $\log_2$ fold change (FC) (x-axis) and $-\log_{10}$ P values (y-axis) of the cytoplasmic proteome of the (**d**) erastin-, (**e**) RSL3-, (**f**) DAT-treated and nontreated cells. Proteins with significant increase after treatment were depicted in red, and those significantly decreased after erastin treatment were depicted in blue. Statistical analysis was performed with two-sided Student's t-test (BH adjusted P values) from three biological replicates.

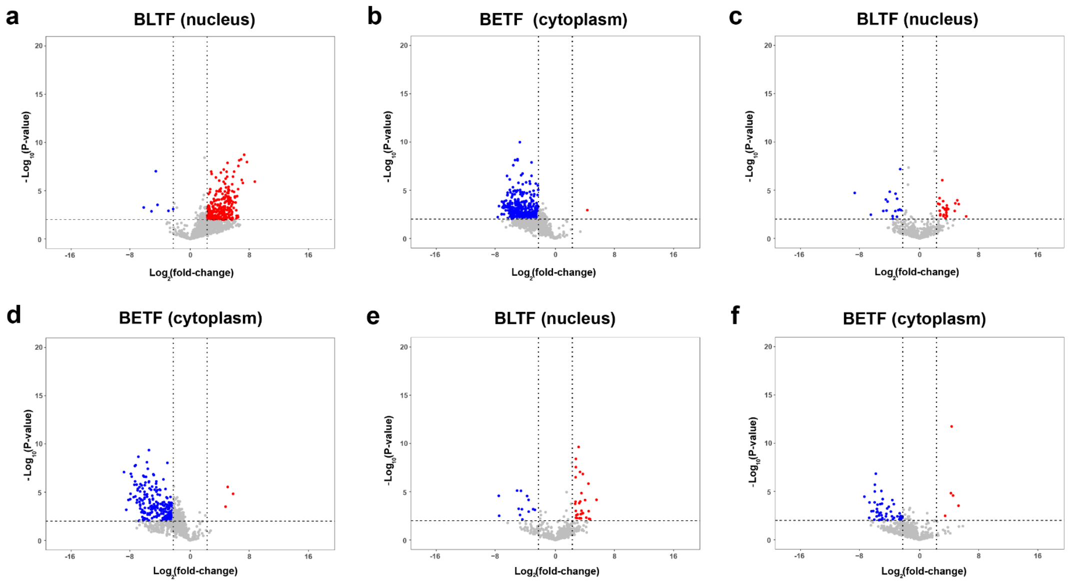

Figure S13. Quantitative analysis of RBPs in nuclear/cytoplasmic RNPs in cells treated with or without ferroptosis inducers.

a, b Volcano plots displaying $\log_2$ fold change (FC) (x-axis) and -log P values (y-axis) of the RBPs quantified from the erastin-treated and nontreated cells by (**a**) nucleus-targeting probe BLTF and (**b**) cytoplasm-targeting probe BETF enrichment.

c, d Volcano plots displaying $\log_2$ fold change (FC) (x-axis) and -log P values (y-axis) of the RBPs quantified from the RSL3-treated and nontreated cells by (**c**) nucleus-targeting probe BLTF and (**d**) cytoplasm-targeting probe BETF enrichment.

e, f Volcano plots displaying $\log_2$ fold change (FC) (x-axis) and -log P values (y-axis) of the RBPs quantified from the DAT-treated and nontreated cells by (**e**) nucleus-targeting probe BLTF and (**f**) cytoplasm-targeting probe BETF enrichment.

Significantly increased RBPs after ferroptosis inducer treatment were depicted in red, and significantly decreased RBPs after ferroptosis inducer treatment were depicted in blue. Statistical analysis was performed with two-sided Student's t-test (BH adjusted P values) from three biological replicates.

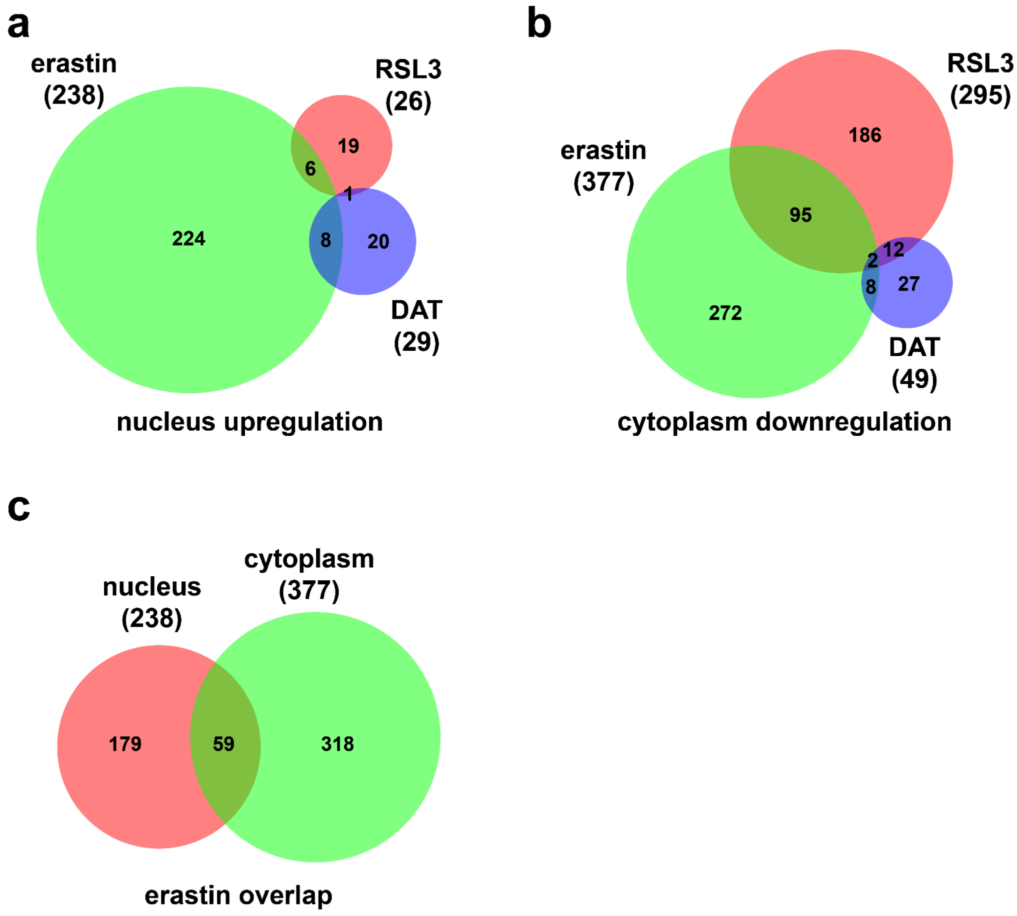

Figure S14. Overlaps of the ferroptosis inducers regulated subcellular RBPs.

a, b Overlap of (**a**) the upregulated RBPs in nuclear RNPs and (**b**) the downregulated RBPs in cytoplasmic RNPs induced by erastin, RSL3 and DAT.

c Overlap of nuclear upregulation and cytoplasmic downregulation RBPs induced by erastin.

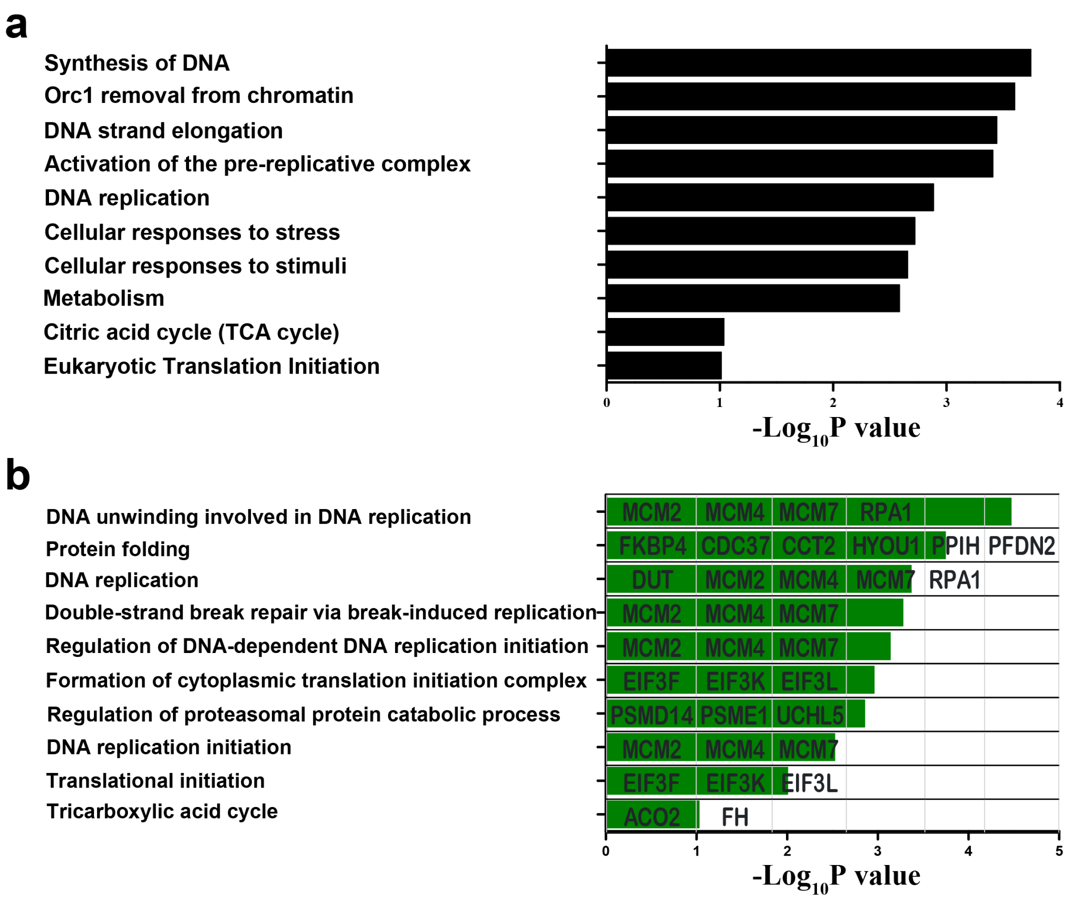

Figure S15. Analysis of the erastin-induced nucleoplasmic translocation candidate RBPs.

a Pathway analysis of the candidates.

b GO biological process analysis of the candidates. Statistical analysis was performed with hypergeometric test (BH adjusted P values).

Table S2. Quantitative analysis of RPS27A and RPL7A identified by subcellular proteome profiling and RNA binding profiling before and after erastin treatment.

Data source	Cellular component	RPL7A (fold change)	RPS27A (fold change)
Subcellular proteome profiling	nucleus	1.96	0.72
	cytoplasm	0.60	1.51
RNA binding enrichment	nucleus	0.97	0.83
	cytoplasm	1.08	1.27

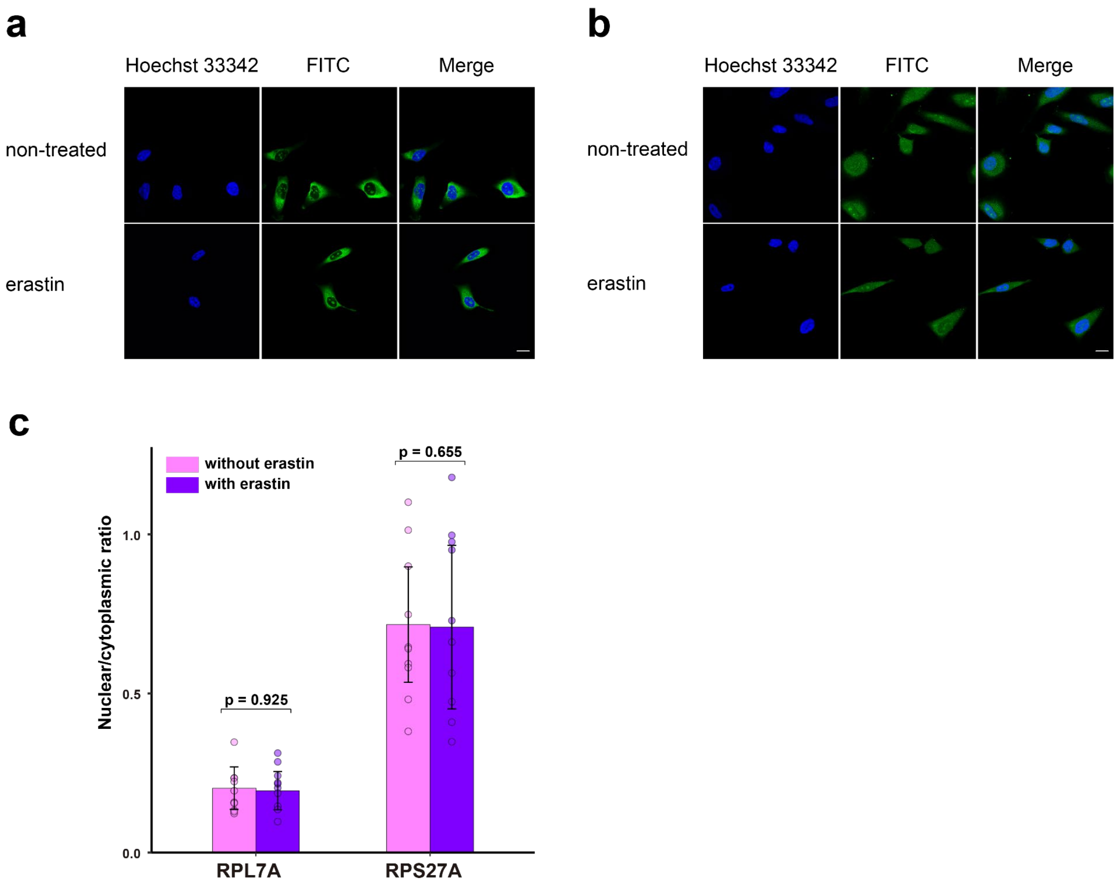

Figure S16. Erastin induced RBPs translocation negative control.

a, b Confocal fluorescent imaging of (**a**) RPL7A and (**b**) RPS27A as negative control for RBPs with/without erastin induction.

c The nuclear/cytoplasmic fluorescence intensity ratio of RPL7A and RPS27A in (**a**) and (**b**). Values are the mean ± S.D. of n=10 cells per condition, two-way ANOVA.

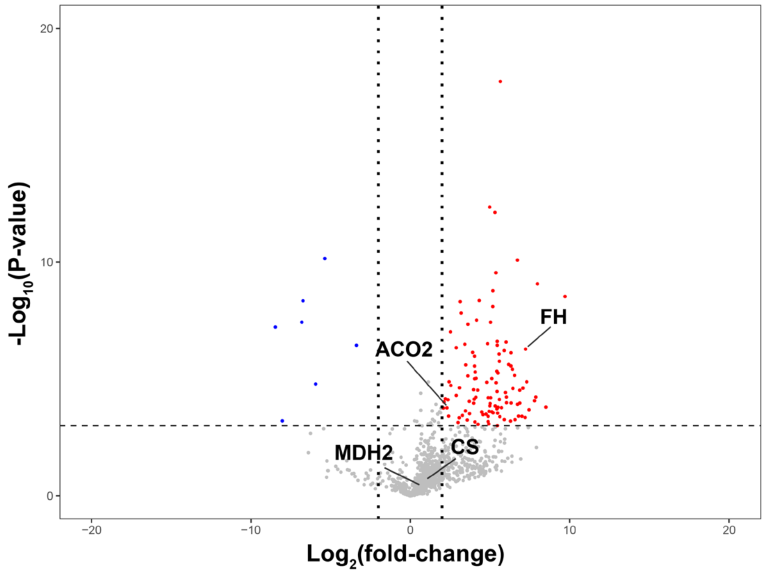

Figure S17. Quantitative analysis of nuclear proteins in cells treated with or without erastin.

Volcano plot displaying $\log_2$ fold change (FC) (x-axis) and $-\log_{10}$ P values (y-axis) of the nuclear proteome of the erastin-treated and nontreated cells. Proteins with significant increase after erastin treatment were depicted in red, and those significantly decreased after erastin treatment were depicted in blue. Statistical analysis was performed with two-sided Student's t-test (BH adjusted P values) from three biological replicates.

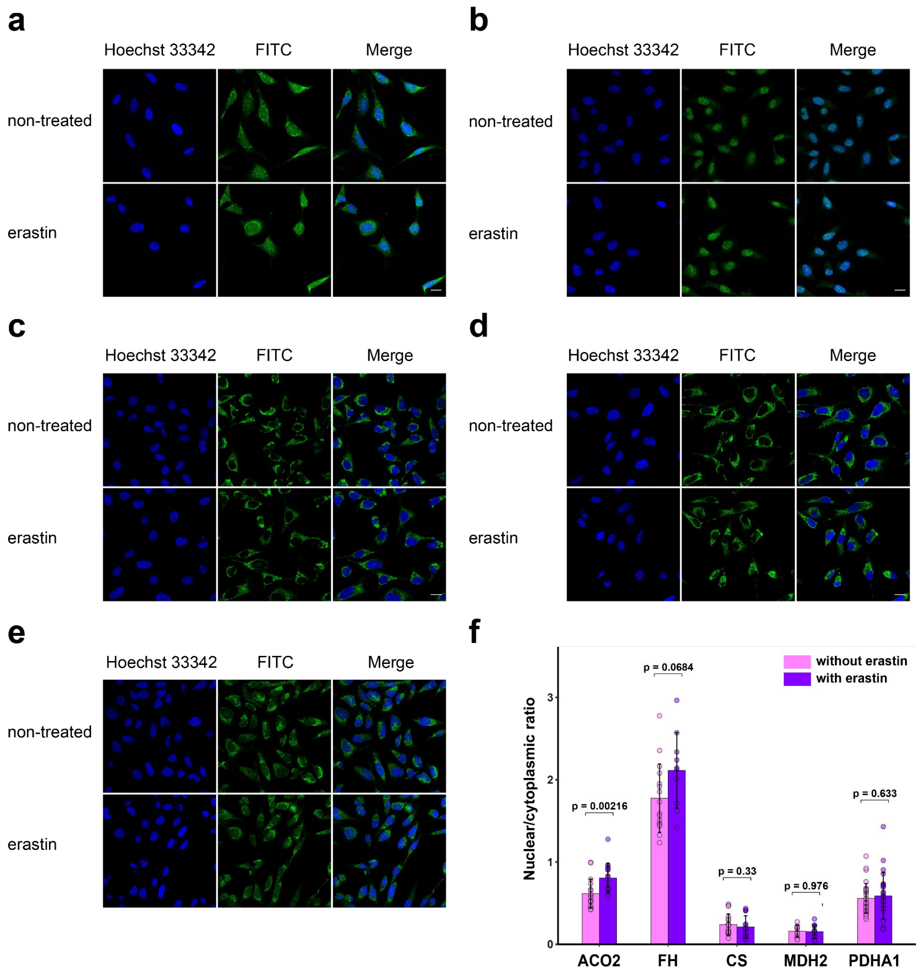

Figure S18. Immunofluorescence imaging of TCA proteins with and without erastin induction.

a-e Immunofluorescence images of (**a**) ACO2, (**b**) FH, (**c**) CS, (**d**) MDH2 and (**e**) PDHA1 with and without erastin induction.

f The nuclear/cytoplasmic fluorescence intensity ratio of ACO2, FH, CS, MDH2 and PDHA1 with and without erastin induction. Values are the mean ± S.D. of n=10 cells per condition, two-way ANOVA.

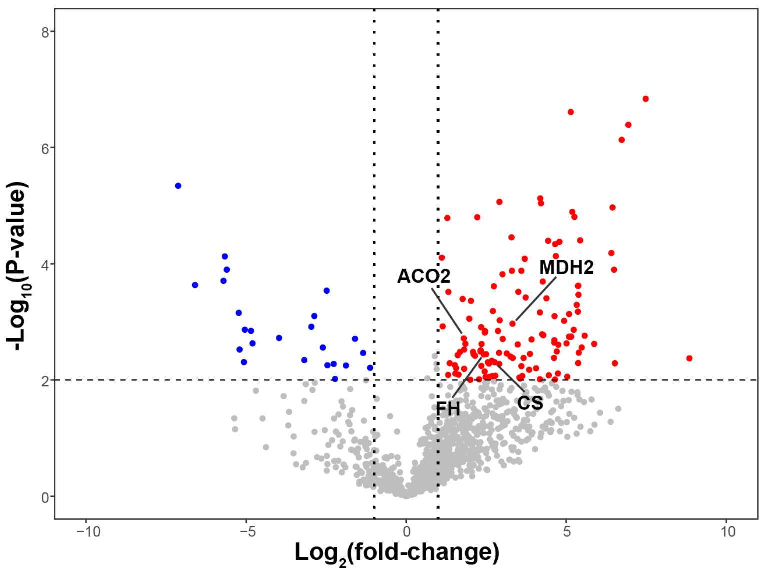

Figure S19. Quantitative analysis of the BLTF probe enriched nuclear RBPs in cells treated with or without erastin after normalization by the corresponding protein abundance variation.

Volcano plot displaying log_2 fold change (FC) (x-axis) and $–log_{10}$ P values (y-axis) of the nuclear RBPs enriched from the erastin-treated and nontreated cells after normalization by the nuclear proteome. Significantly increased nuclear RBPs after erastin treatment were depicted in red, and significantly decreased ones were depicted in blue. Statistical analysis was performed with two-sided Student's t-test (BH adjusted P values) from three biological replicates.

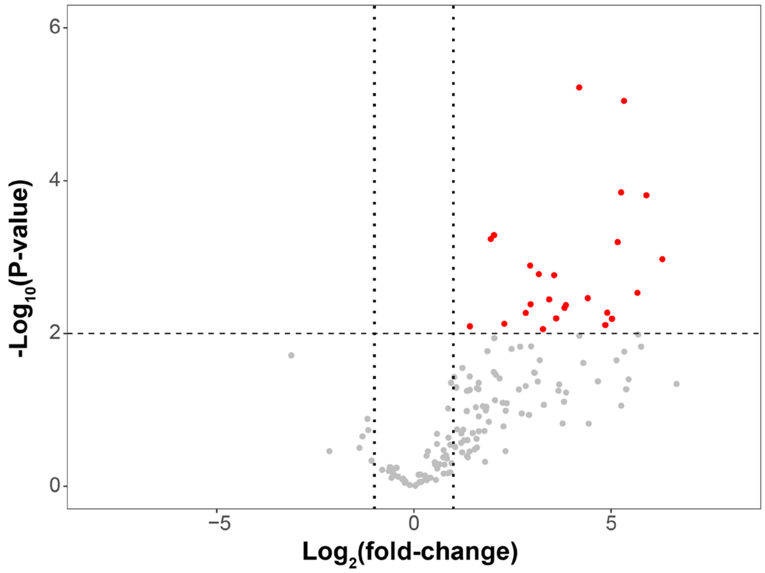

Figure S20. Quantitative analysis of the CA-RBPs in cells treated with or without erastin. Volcano plot displaying $\log_2$ fold change (FC) (x-axis) and $-\log_{10}$ P values (y-axis) of the CA-RBPs quantified from the erastin-treated and nontreated cells. CA-RBPs with significant increase after erastin treatment were depicted in red, and significantly decreased after erastin treatment were depicted in blue. Statistical analysis was performed with two-sided Student's t-test (BH adjusted P values) from three biological replicates.
