# Supplementary material for: Nuclear and cytoplasmic specific RNA binding proteome enrichment and its changes upon ferroptosis induction
Source: Nat Commun. 2024 Jan 29;15:852. doi: 10.1038/s41467-024-44987-9 (PMC10825125; doi:10.1038/s41467-024-44987-9)
Supplement: Supplementary file 2 — Reporting summary [file 41467_2024_44987_MOESM2_ESM.pdf]

nature portfolio

Corresponding author(s): Weijie Qin

Last updated by author(s): Dec 21, 2023

Reporting Summary

Nature Portfolio wishes to improve the reproducibility of the work that we publish. This form provides structure for consistency and transparency in reporting. For further information on Nature Portfolio policies, see our Editorial Policies and the Editorial Policy Checklist.

Statistics

For all statistical analyses, confirm that the following items are present in the figure legend, table legend, main text, or Methods section.

n/a	Confirmed	
☐	☒	The exact sample size (*n*) for each experimental group/condition, given as a discrete number and unit of measurement
☐	☒	A statement on whether measurements were taken from distinct samples or whether the same sample was measured repeatedly
☐	☒	The statistical test(s) used AND whether they are one- or two-sided<br>*Only common tests should be described solely by name; describe more complex techniques in the Methods section.*
☒	☐	A description of all covariates tested
☐	☒	A description of any assumptions or corrections, such as tests of normality and adjustment for multiple comparisons
☐	☒	A full description of the statistical parameters including central tendency (e.g. means) or other basic estimates (e.g. regression coefficient) AND variation (e.g. standard deviation) or associated estimates of uncertainty (e.g. confidence intervals)
☐	☒	For null hypothesis testing, the test statistic (e.g. *F*, *t*, *r*) with confidence intervals, effect sizes, degrees of freedom and *P* value noted<br>*Give P values as exact values whenever suitable.*
☒	☐	For Bayesian analysis, information on the choice of priors and Markov chain Monte Carlo settings
☒	☐	For hierarchical and complex designs, identification of the appropriate level for tests and full reporting of outcomes
☒	☐	Estimates of effect sizes (e.g. Cohen's *d*, Pearson's *r*), indicating how they were calculated

Our web collection on statistics for biologists contains articles on many of the points above.

Software and code

Policy information about availability of computer code

Data collection	LC-MS/MS data collection was achieved by Thermo Xcalibur software v4.3. The RNA-seq was performed on an Illumina HiSeq 2500 platform.
Data analysis	The analysis of LC-MS/MS data was performed in MaxQuant (version 2.4.2.0). GO and pathway analyses were conducted using Funrich Version 3.1.3 and Reactome. Protein domain analysis was performed using SMART (http://smart.embl-heidelberg.de/) against the PFAM and SMART domain databases. Global protein sequence features were computed using the R (Version 4.0.3) package 'peptides' with the scales 'Kyte-Doolittle' for hydrophobicity and 'EMBOSS' for isoelectric point. The intrinsic disorder of proteins was derived using IUPred (https://iupred2a.elte.hu/). Disordered amino acid residues were defined by an IUPred score of 0.4, and the fraction of disordered amino acid residues was computed for each protein. The percentage of positively charged amino acids was calculated by Python (Version 3.8).

For manuscripts utilizing custom algorithms or software that are central to the research but not yet described in published literature, software must be made available to editors and reviewers. We strongly encourage code deposition in a community repository (e.g. GitHub). See the Nature Portfolio guidelines for submitting code & software for further information.

Data

Policy information about availability of data

All manuscripts must include a data availability statement. This statement should provide the following information, where applicable:

- Accession codes, unique identifiers, or web links for publicly available datasets
- A description of any restrictions on data availability
- For clinical datasets or third party data, please ensure that the statement adheres to our policy

The mass spectrometry proteomics data have been deposited to the ProteomeXchange Consortium (http://proteomecentral.proteomexchange.org) via the iProX partner repository with the dataset identifier PXD033927 under accession code hb5N (https://www.iprox.cn/page/PSV023.html;?url=1684305985768tMLc). All RNA-seq data used in this manuscript have been deposited in Gene Expression Omnibus (www. ncbi.nlm.nih.gov/geo). The data about the type and relative distribution of the isolated RNAs were deposited under accession number GSE205553 (https://www.ncbi.nlm.nih.gov/geo/query/acc.cgi?acc=GSE205553).

Research involving human participants, their data, or biological material

Policy information about studies with human participants or human data. See also policy information about sex, gender (identity/presentation), and sexual orientation and race, ethnicity and racism.

Reporting on sex and gender	NA
Reporting on race, ethnicity, or other socially relevant groupings	NA
Population characteristics	NA
Recruitment	NA
Ethics oversight	NA

Note that full information on the approval of the study protocol must also be provided in the manuscript.

Field-specific reporting

Please select the one below that is the best fit for your research. If you are not sure, read the appropriate sections before making your selection.

☒ Life sciences ☐ Behavioural & social sciences ☐ Ecological, evolutionary & environmental sciences

For a reference copy of the document with all sections, see nature.com/documents/nr-reporting-summary-flat.pdf

Life sciences study design

All studies must disclose on these points even when the disclosure is negative.

Sample size	No sample size calculation was performed. Each MS experiment used of a 150-mm dish of HeLa cells. Immunofluorescence used a 35 mm glass-bottom culture dish of HeLa cells for each sample.
Data exclusions	No data were excluded from the analyses.
Replication	All experiments were performed in three independent biological replicates. All attempts at replication were successful.
Randomization	Allocation was random.
Blinding	We were blinded to group allocation.

Reporting for specific materials, systems and methods

We require information from authors about some types of materials, experimental systems and methods used in many studies. Here, indicate whether each material, system or method listed is relevant to your study. If you are not sure if a list item applies to your research, read the appropriate section before selecting a response.

Materials & experimental systems

n/a	Involved in the study	
☐	☒	Antibodies
☐	☒	Eukaryotic cell lines
☒	☐	Palaeontology and archaeology
☒	☐	Animals and other organisms
☒	☐	Clinical data
☒	☐	Dual use research of concern
☒	☐	Plants

Methods

n/a	Involved in the study	
☒	☐	ChIP-seq
☒	☐	Flow cytometry
☒	☐	MRI-based neuroimaging

Antibodies

Antibodies used

Anti-Lamin A/C (1 ug/ml, ab238303, Abcam), Anti-beta tubulin (1:500 dilution, ab108342, Abcam), Anti-Histone H3 (1:2000 dilution, ab176842, Abcam), Anti-EIF5 (1:200 dilution, ab170915, Abcam), Anti-FXR1 (1:500 dilution, ab155124, Abcam), Anti-ECHS1 (1:500 dilution, ab228631, Abcam), AntI-RPL7A (1:50 dilution, 15340-1-AP, Proteintech), Anti-RPS27A (1:50 dilution, 14946-1-AP, Proteintech), Anti-ACO2 (1:1000 dilution, ab228923, Abcam), Anti-FH/Fumarase (1:1000 dilution, ab233393, Abcam), Anti-Citrate Synthase (1:50 dilution, 14309S, cell signaling), Anti-MDH2 (2 ug/ml, ab110317, Abcam), Anti-PDHA1 (1:500 dilution, ab168379, Abcam), CoraLite488-conjugated Goat Anti-Rabbit IgG(H+L) (1:500 dilution, SA00013-2, Proteintech), HRP-conjugated Affinipure Goat Anti-Mouse IgG(H+L) (1:100 dilution, SA00001-1, Proteintech).

Validation

Validation of these antibodies could be found on the websites of the suppliers.

Eukaryotic cell lines

Policy information about cell lines and Sex and Gender in Research

Cell line source(s)

HeLa cells were purchased form ATCC.

Authentication

HeLa cells were authenticated by ATCC using short tandem repeat (STR) profiling.

Mycoplasma contamination

All cell lines tested negative for mycoplasma contamination.

Commonly misidentified lines (See ICLAC register)

No commonly misidentified cell lines were used.
