# Supplementary material for: Nuclear and cytoplasmic specific RNA binding proteome enrichment and its changes upon ferroptosis induction
Source: Nat Commun. 2024 Jan 29;15:852. doi: 10.1038/s41467-024-44987-9 (PMC10825125; doi:10.1038/s41467-024-44987-9)
Supplement: Supplementary file 3 — Description of Additional Supplementary Files [file 41467_2024_44987_MOESM3_ESM.pdf]

Description of Additional Supplementary Files Document

Supplementary Data 1

Datasets of RBPs identified by subcellular targeting probes. Tables include the RBPs identified by (1) the six probes, (2) the three nucleus-targeting probes, (3) the three cytoplasm targeting prbes, (4) BLF, (5) BLTF, (6) BL3F, (7) BETF, (8) BELF, (9) BLEF, and (10) list of RBPs identified by previous RBP profiling methods.

Supplementary Data 2

Datasets of differential analysis of RBPs identified by BLTF and BETF. Tables include (1) the differential analysis of RBPs identified by BLTF and BETF and (2) co-identified RBPs.

Supplementary Data 3

Datasets of differential analysis of subcellular proteome between the ferroptosis inducer treated and non-treated cells. Tables include the differential analysis of nuclear proteome between (1) erastin, (2) RSL3 and (3) DAT treated and non-treated cells, and cytoplasmic proteome between (4) erastin, (5) RSL3 and (6) DAT treated and non-treated cells.

Supplementary Data 4

Datasets of differential nuclear and cytoplasmic RBPs between the ferroptosis inducer treated and non-treated cells. Tables include the datasets of BLTF identified (1) erastin upregulated, (2) erastin downregulated, (3) RSL3 upregulated, (4) RSL3 downregulated, (5) DAT upregulated and (6) DAT downregulated nuclear RBPs compared with non-treated cells, and BETF identified (1) erastin upregulated, (2) erastin downregulated, (3) RSL3 upregulated, (4) RSL3 downregulated, (5) DAT upregulated, (6) DAT downregulated cytoplasmci RBPs compared with non-treated cells and (7) Nucleoplasmic 59 translocation candidates.

Supplementary Data 5

Dataset of the protein-protein interaction (PPI) network. Tables include the nodes and edges with their interactors of the PPI network.
